# Advancing host-directed therapy for tuberculosis: new therapeutic insights from the *Toxoplasma gondii*

**DOI:** 10.15698/mic2017.03.565

**Published:** 2017-03-02

**Authors:** Chul-Su Yang

**Affiliations:** 1 Department of Molecular and Life Science, Hanyang University, Ansan 15588, S. Korea.; 2 Department of Bio-nanotechnology, Hanyang University, Seoul, 04673, S. Korea.

**Keywords:** Toxoplasma gondii GRA7, macrophages, protein-protein interactions, Mycobacterium tuberculosis

## Abstract

Tuberculosis (TB) drug-development strategies, a wide range of candidate host-directed therapies (HDT)s-including new and repurposed drugs, biologics, and cellular therapies-have been proposed to accelerate eradication of infection and overcome the problems associated with current treatment regimens. By investigating the interaction between macrophages and the intracellular parasite *Toxoplasma gondii* (*T. gondii*), we uncovered that infection-induced signaling pathways suggest possibilities for the development of novel therapeutic modalities for TB that target the intracellular signaling pathways permitting the replication of *Mycobacterium tuberculosis* (MTB).

Previously, we uncovered that *T. gondii* dense granule antigen (GRA) 7/MyD88-dependent NF-κB activation is essential for the activation of TNF receptor-associated factor 6 (TRAF6) and reactive oxygen species generation, and enhances the release of inflammatory mediators. We also found that GRA7 stimulation led to physical and functional associations between GRA7 and TRAF6, resulting in crucial protective efficacy against *T. gondii* infection *in vivo*. However, its exact role and how it regulates host innate immune responses have not been fully explained. It remains to be seen whether GRA7 targeting can be used as a therapeutic strategy for infectious diseases. In our recent study (Koh *et al*., PLoS Pathog. 2017 Jan 26;13(1):e1006126), we identified that GRA7 interacts with host proteins involved in antimicrobial host defense mechanisms as a therapeutic strategy for tuberculosis. We underscore a previously unrecognized role of GRA7 in modulating antimicrobial host defense mechanism during mycobacterial infection.

TB is a global health problem and at least one-third of the world’s population is infected with MTB. MTB is a successful pathogen that enhances its own intracellular survival by inhibiting inflammation and arresting phago-lysosomal fusion. Recent studies have revealed the intracellular signaling pathways that govern the outcome of the innate immune response to mycobacteria infection and antibacterial defense.

In our recent study, we further investigated the intracellular regulatory network of *T. gondii* GRA7-induced of apoptosis-associated speck-like protein containing a carboxy-terminal CARD (ASC), phospholipase D (PLD) 1, and protein kinase C (PKC) α signaling pathways to help identify novel therapeutic modalities for TB. We found that the PKCα-mediated phosphorylation of *T. gondii *GRA7 is essential for the interaction between GRA7 and ASC or PLD1, which contributes to antimicrobial defense against MTB (Fig. 1). Specifically, we found that (1) PKCα specific phosphorylation of Ser52 and Ser135 of GRA7 *in vitro *and* in vivo* was functionally required for ASC and PLD1 interactions with GRA7, respectively, (2) GRA7 was a novel substrate of PKCα, (3) the N-terminal of GRA7 (GRA7-I) was sufficient for interaction with the PYD domain of ASC in mitochondria, leading to ASC oligomerization and inflammasome activation, and subsequent antimicrobial activity, (4) GRA7-III interacted with the PX domain of PLD1 in cytosol, facilitating its enzyme activity, phago-lysosomal biogenesis, and subsequent antimicrobial activity, (5) GRA7-I and -III-dependent host protective effects against MTB infection were demonstrated *in vivo*, and (6) a phosphomimetic mutant of GRA7 that constitutively activated GRA7 ‘rescued’ PKCα deficiency both *in vitro* and *in vivo*. Collectively, these observations indicate that *T. gondii* GRA7-mediated HDTs leading to an antimicrobial response, as a novel host defense mechanism may provide a unique opportunity for urgently needed therapeutic intervention strategies for TB and other infectious diseases.

**Figure 1:**
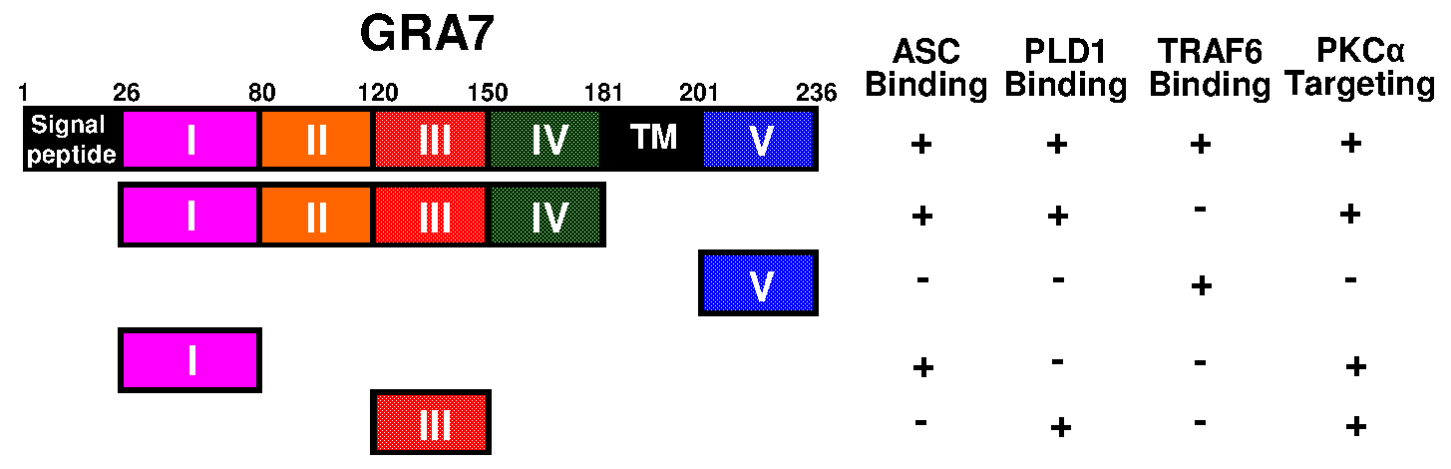
FIGURE 1: Summary of the interactions of GRA7 and its mutants with ASC, PLD1, TRAF6, and PKCα.

Recent developments in TB drug-development strategies (including new and repurposed antimicrobials and host-directed drugs) have produced new regimens to shorten treatment duration, improve outcomes of TB treatment such as: prevent resistance, reduce lung injury by promoting autophagy, antimicrobial peptide production, and other macrophage effector mechanisms, as well as inhibiting mechanisms causing lung inflammation and matrix destruction.

**Figure 2:**
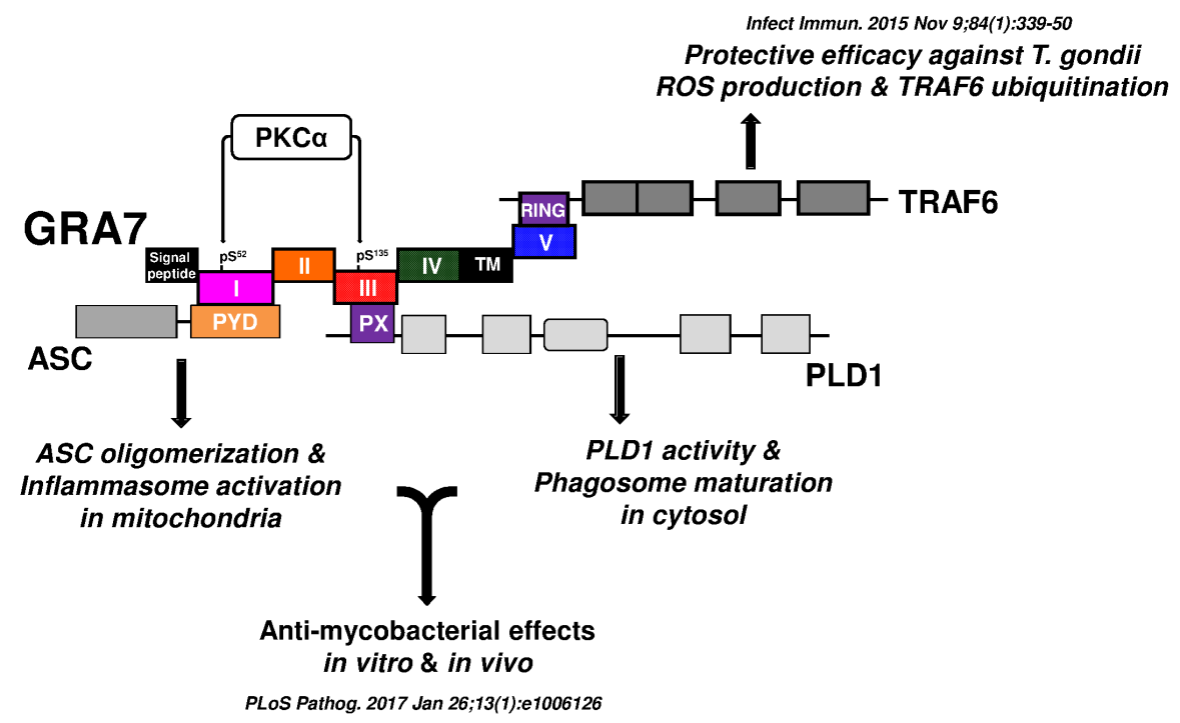
FIGURE 2: Advancing host-directed therapy for tuberculosis: new therapeutic insights from the *T. gondii* GRA7. Schematic model for the roles of GRA7 and GRA7-mediated regulatory pathways against intracellular pathogens such as *Mycobacteria *and *T. gondii.*

We showed that GRA7 protein interacted with a number of host cell proteins including enzymes, and a broad spectrum of structural and functional subcellular organellar proteins revealing a new facet of the role of GRA7 in the regulation of innate host immune responses. Furthermore, GRA7-I and -III play fine-tuning roles in the activation of HDTs and innate immune machineries through direct binding with ASC or PLD1 and may provide a unique opportunity for urgently needed therapeutic interventions against TB.

The rGRA7 have a function of biologicals as potential therapeutics. However, these rGRA7 do not fulfil the requirements of direct anti-mycobacterial agent, which represent feasible alternatives to conventional chemotherapy to TB, due to the still unclear specificity and selectivity does not enable linking the effects of rGRA7s to host immune systems, as well as limitation of animal experimental model, unknown off-target effects, pharmacokinetics, safety data, and their potential feasibility for *in vivo* proof-of-concept studies. Further analyses are required to find out whether rGRA7s can be translated to the *in vivo* situation or be observed in the presence of physiological condition to patient with TB.

Nevertheless, our observations reveal a new role for GRA7 in regulating innate immune responses in host protective immunity. Our findings establish proof of concept for HDT strategies that manipulate host GRA7-mediated immune networks and represent feasible alternatives to conventional chemotherapy to TB. Further studies are needed to develop more effective GRA7-based potential therapeutic targets and to understand how GRA7 regulates host defense strategies against TB and other infectious diseases.

